# Cardiac Rehabilitation and Exercise Prescription in Symptomatic Patients with Non-Obstructive Coronary Artery Disease—a Systematic Review

**DOI:** 10.1007/s11936-018-0667-2

**Published:** 2018-08-18

**Authors:** Christine K. Kissel, Dimitra Nikoletou

**Affiliations:** 1 Department of Cardiology, University Heart Center, University Hospital Zurich, Zurich, Switzerland; 2 Msc Sports Cardiology, Cardiology Clinical Academic Group, St George’s, University of London, London, UK; 3 Emergency, Cardiovascular and Critical Care Research Group, Faculty of Health, Social Care and Education (Joint Faculty), Kingston University & St George’s University of London, London, UK

**Keywords:** Microvascular angina, Coronary artery disease, Exercise, Rehabilitation

## Abstract

**Purpose of review:**

Non-obstructive coronary artery disease (NOCAD) on coronary angiography is a common finding in patients with stable angina. Angina in NOCAD patients is thought to be caused by endothelial dysfunction of the epicardial coronary arteries and/or the microvasculature. Treatment is empiric, and 30% of patients remain symptomatic in spite of therapy. It is well known that physical exercise can improve endothelial function. The goal of this review was to assess the current literature on effects of physical exercise in NOCAD patients with angina. Therefore, a literature search was conducted to March 13, 2018 using the following search terms: syndrome X, microvascular angina, non-obstructive coronary artery disease and exercise training, cardiac rehabilitation, endothelial function. All original publications were included which examined the effect of a cardiac rehabilitation (CR) program or exercise training (ET) on patients with angina and NOCAD.

**Recent findings:**

Eight studies, of which four were randomized controlled studies, examined 218 participants, 162 in an intervention and 56 in control groups. Most patients were women (97.7%). Exercise programs varied from 8 weeks to 4 months at moderate intensity and some included relaxation therapy. The studies examined the effect of CR on exercise capacity, quality of life (QoL), and perfusion defects. CR increased exercise capacity, oxygen uptake, symptom severity, and QoL. Myocardial perfusion improved.

**Summary:**

CR appears to be beneficial in symptomatic patients with NOCAD, improving exercise capacity and QoL and reducing severity of symptoms and myocardial perfusion defects. Data is limited to a small number of predominantly female patients. Further larger trials are warranted to determine the optimal rehabilitation protocols and define its long-term benefits.

## Introduction

Non-obstructive coronary artery disease (NOCAD) is often found on coronary angiograms performed in patients with stable angina, with up to 60% of women having NOCAD [1••]. Angina in the context of NOCAD appears to be caused by endothelial dysfunction at the epicardial and/or microvascular level [2•]. Albeit a heterogonous population, the consensus is that inappropriate coronary vasodilator capacity is the common denominator [2•]. Patients previously diagnosed with “cardiac syndrome X” (CSX) also belong to this entity and are now classified as microvascular angina [3].

Numerous studies indicate an increased risk of cardiovascular events in these patients and high costs due to frequent rehospitalization, repeat diagnostic procedures, and medical therapy [1••, 4, 5]. Approximately one third of patients remain symptomatic in spite of therapeutic interventions, which are often empirical [5, 6•]. NOCAD angina is associated with depression and decreased quality of life (QoL) [7]. The duration and intensity of symptoms are independent predictors of reduced QoL in women with NOCAD [8].

It is well known that exercise improves endothelial function in patients with coronary artery disease (CAD) [9] and heart failure [10]. Furthermore, exercise training (ET) is a fundamental part of cardiac rehabilitation (CR) which has been repeatedly shown to reduce cardiovascular mortality and rehospitalization rates and improve psychological well-being and risk factor control in patients with CAD [11].

The goal of the systematic review was to appraise available literature on CR in patients with NOCAD/microvascular angina and to assess whether there is evidence suggestive of a potential benefit in these patients with respect to endothelial function, cardiovascular outcomes, symptom control, QoL, and exercise capacity.

## Methods

Studies were identified by conducting systematic searches of PubMed, EMBASE, and the Cochrane Library up until March 13, 2018. Searches included a mix of MeSH and free text terms related to syndrome X, microvascular angina, non-obstructive coronary artery disease, exercise training, cardiac rehabilitation, and endothelial function. Additionally, systematic reviews and reference lists of papers were hand searched for additional studies. All clinical trials with ET/CR in symptomatic patients with NOCAD were included. The selection was not limited to randomized controlled trials because of the paucity of data in this field. Articles written in languages other than English were excluded.

## Results

Eight original articles included 218 patients, of which 162 underwent an intervention, and 56 served as controls. The majority of patients in these studies were women (97.7%). Inclusion criteria were ongoing exertional chest pain (CP) without significant CAD on CT scan or coronary angiography, and the majority of studies required a positive exercise stress test or perfusion scan. The studies were conducted in five different countries (UK, Poland, Sweden, Brazil, Iran). Four of the studies were randomized, controlled clinical studies [12–15].

The first study on physical training in patients with CSX was published by Eriksson et al. in 2000 [13]. The authors randomized women into three groups which consisted of 8-week body-awareness (BA) program followed by 8 weeks of ET (total of 16 weeks), 8 weeks of ET alone, or a no-intervention control group [13]. Exercise training entailed cycle ergometer training three times a week for 30 min at 50% of peak work rate, which was determined at the beginning of the study. Endpoints were exercise capacity, urinary excretion of catecholamines and cortisol as an indicator for “negative stress,” adenosine sensitivity test as a measure for pain sensitivity, and assessment of flow-mediated dilation (FMD) as an indicator of endothelial function. The authors were able to show that ET but not BA improved the exercise capacity by 36% and oxygen uptake by 26%. Time to pain onset changed significantly after ET, but not after sole BA training. Pain sensitivity was not affected by BA or ET. Cortisol levels decreased after BA, whereas catecholamine levels remained unchanged. There was a trend towards an improved endothelial function in the ET cohort [13].

The same study group examined 21 women in 2002 [12]. They randomized women into three groups (A, ET; B, Jacobson relaxation therapy, autogenous training; C, control group). ET consisted of the same protocol as in the prior study [13]. QoL and coping capacity were examined by three different questionnaires (SOC, SCI-93, SIP) [12]. Health-related QoL improved in both the ET and the relaxation therapy groups, which was not associated with improved coping strategies [12].

In 2008, Asbury et al. published data on 64 women with diagnostic criteria for CSX, which were randomized into two groups [15]. One group underwent a standard 8-week group-based phase III CR program, which included an 80-min hospital-based circuit training once a week with the aim to exercise at 60–75% of their age-predicted heart rate reserve [15]. This was complemented by a home exercise session once a week. The other half had an 8-week symptom-monitoring program only. The patients’ physical capacity was assessed by progressive shuttle walk (PSW) test. Each patient was asked to complete a symptom-monitoring diary daily and to fill out five different questionnaires to assess their psychological health (Health Anxiety Questionnaire, Hospital Anxiety and Depression Scale, SF-36, Cardiac Anxiety Questionnaire, Demographic Information Scale) at the beginning and at the end of the study. Compared to controls, women in the ET group showed significantly reduced symptom severity and psychological morbidity and were able to walk a longer distance in the PSW test [15].

Feizi et al. examined 40 women with CSX in Iran in 2012 [14]. Patients were randomized into four groups ((1) control, (2) CR only, (3) progressive muscle relaxation (PMR) only, (4) CR + PMR). The PMR group was instructed to perform Jacobson’s method for 15 min per day at home. The CR group performed a home-based phase III program that consisted of an initial 25-min walk, which gradually increased to 40 min at 60–65% of maximum heart rate, three times per week, for 8 weeks. The fourth group combined PMR and CR. Outcomes were assessed by the SF-36 questionnaire. In all three intervention groups, QoL improved. However, the combination of CR and PMR led to the most pronounced improvement of QoL scores [14].

Carvalho et al. published data from Brazil in 2014 [16]. Patients were included with exertional CP, normal coronary angiography, and presence of ≥ 2 myocardial segments with reversible perfusion defects (RPDs) identified by SPECT myocardial perfusion scan. This was the only study which included men (5/12). Patients underwent a 4-month rehabilitation program that consisted of three sessions per week of treadmill training at 60–85% of peak VO_2_. The program also included global stretching (5 min), resistance exercise (10 min), and relaxation (5 min). At the end of the program, SPECT was repeated, as well as application of the SF-36 questionnaire. The authors showed a 14% increase in oxygen uptake and were able to show a significant reduction in myocardial ischemia on SPECT (SDS, summed difference score). There was a significant improvement of the SF-36 scores in all categories but general health status [16]. In a further study, Carvalho et al. compared the same patient population with a healthy control group in regard to left ventricular ejection fraction (LVEF) at peak exercise on radionuclide ventriculography [17]. Compared to healthy controls, the LVEF in patients with MA did not rise significantly at peak exertion and this did not improve with exercise training over 4 months [17].

The latest studies were published by Szot et al. from Poland who examined 55 women with CSX and RPDs on SPECT [18]. Patients underwent an exercise program for 3 months with ET scheduled three times a week for 90 min (30-min warm-up, 30-min bicycle ergometer, 30-min relaxation). The training intensity was increased from 70 to 100 W after 4 weeks and to 120 W after another month. The intensity was reduced if 80% of maximum heart rate was achieved. After 3 months, the Ferrans & Powers Quality of Life Index questionnaire was repeated, as well as SPECT. The authors demonstrated a 20% increase in exercise capacity, as well as an improved perfusion on SPECT during exercise. There was a weak correlation between improved SDS and QoL [18]. The same study group published another paper with the same cohort, showing an increase in exercise capacity, improved systolic and diastolic blood pressure, reduced body mass index (BMI), and improved SDS [19].

**Fig. 1:**
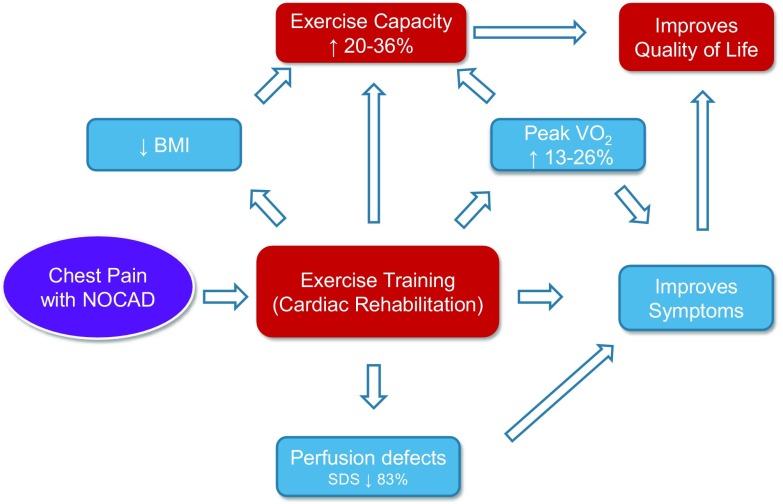
Beneficial effects of cardiac rehabilitation in patients with NOCAD. BMI, body mass index; SDS, summation difference score; NOCAD, non-obstructive coronary artery disease, VO_2_, oxygen uptake.

In summary, the duration of the exercise programs varied from 8 weeks to 4 months, with most of the programs lasting 8 weeks (Table 1). The majority of interventions were hospital-based. Only Feizi et al. prescribed a solely home-based program, whereas Asbury et al. used a mix of hospital- and home-based sessions. Most of the exercise programs performed three training sessions per week that lasted at least 30 min, only Asbury et al. prescribed three sessions per week of longer duration (Table 1). The minimum training time per week was 90 min. The training mostly consisted of cycle ergometer but also included walking for the home-based program. Exercise intensity varied between the different studies but most authors tried to achieve a moderate intensity, guided by percentage of maximum heart rate [14, 18, 19], heart rate reserve [15], peak VO_2_ [16], or peak work rate [12, 13] (Table 1). Figure 1 summarizes the positive effects of CR in symptomatic patients with NOCAD. ET improved exercise capacity in all studies, albeit to a varying extent. In addition, ET improved QoL and symptom severity in most studies. The combination of relaxation therapies with ET or relaxation therapy alone appeared to have a positive impact on QoL parameters in three studies.

**Table 1:** Overview of clinical studies

Study	Cohort/inclusion criteria	Intervention	Duration of program	Outcome measures	Results
Eriksson et al. 2000 [13] Sweden Randomized	26 women CP, pos. EST, nl angio	A (8): body awareness 8 wks, 2×/wk, then cycle ergometer 3×/wk for 8 wks (50% of peak WR) for 30 min B (8): cycle ergometer 3×/wk for 30 min (50% of peak WR) for 8 wks C (10): control	8 weeks	Cycle ergometer: Peak work rate Peak VO_2_ (l/min) Pain onset 24-h urinary excretion: epinephrine norepinephrine cortisol Pain perception (adenosine sensitivity) Endothelial function: Flow-mediated dilation NTG mediated	Group A + B: A: 36%↑ B 31%↑ A: 26%↑↑ time to pain ↑ C ns ns ns ↓ (A + B) unchanged ns ns
Tynne-Lenne et al. 2002 [12] Sweden Randomized	21 women CP, angio nl, pos. EST	A. ET (7) cycle ergometer 3×/wk for 8 wks (50% of peak WR) for 30 min B. Relaxation training (7); PMR Jacobson, autogenous training C. Control (7)	8 weeks	Cycle ergometer: peak work rate (W) Peak VO_2_ (l/min) 6-min walking: walking distance QoL: Questionnaires: Sense of Coherence (SOC) Stress Crisis Inventory Sickness Impact Profile	A: 31% ↑, B, C: ns A: 15% ↑, B,C: ns A: 5.7% ↑ Coping capacity (SOC): ns (A, B, C) health-related QoL ↑ (A+ B)
Asbury et al. 2008 [15] UK Randomized	64 women angio nl, EST pos., CP+	A. (32) phase III CR exercise program- 1 80-min hospital-based CR class 60–75% of age-predicted HRR); 1 home exercise program B. (32) monitor only	8 weeks	BMI, DBP Progressive shuttle walk: Distance Attained level QoL: Health Anxiety Questionnaire Hospital Anxiety and Depression Scale SF-36 Cardiac Anxiety Questionnaire Demographic Information Scale	A: improved 29.7%↑ A 26.4%↑ A A: psychological morbidity↓, symptom severity↓, QoL↑,
Feizi et al. 2012 [14] Iran Randomized	40 women CP, pos. EST and normal angiography	A (7): controls B (11): CR phase III 3×/week; warm-up 60–65% of max heart rate, at first 25 min with increase to 40 min, cool down; @ home, monitoring by phone C (11): PMR (15′ daily at home) D (11): CR + PMR	8 weeks	QoL: SF-36	A-D: QoL ↑ B+ D: physical functioning ↑ D: physical role, total QoL better
Carvalho et al. 2015 [16] Brazil	12 patients (7 women) Ischemia on SPECT, normal angiography, CP	3×/week 1 h 60–85% of VO_2_ peak on CPT: 5′ global stretching, 5′ treadmill warm-up, 30′ physical conditioning, 5′ cool down, 10′ resistance exercise, 5′ relaxation	4 months	Exercise Treadmill: Peak VO_2_ (ml/kg/min) SPECT: SDS SRS SSS QoL: SF-36	14%↑ SDS 71% ↓ SRS ns SSS 72% ↓ All categories significantly improved except general health status
Szot et al. 2015 [18], Szot et al. 2016 [19] Poland	55 women CP, pos. SPECT and exclusion of CAD (CCT/angiography)	90 min, groups of 6; 3×/wk; 30-min warm-up 30-min bicycle ergometer at 70, 100, 120 W (increase after 4 wks) Relaxation exercises Max. 80% of max HR (treadmill test)	12 weeks	Physical: BMI, SBP, DBP, HR Exercise treadmill: METs SPECT: SDS SRS SSS QoL: Ferrans & Powers Quality of Life Index	Improved 24.7%↑ SDS sum ↓72% SRS sum ns SSS sum ↓35% Improved (total + related to cardiac sx)
Carvalho et al. 2017 [17]	12 patients (7 women), 15 controls (6 women) exclusion of CAD on angio, ischemia on SPECT (≥ 2 segments)	60 min, 3×/wk 60–85% of VO_2_ peak 5′ global stretching, 5′ treadmill warm-up, 30′ physical conditioning, 5′ cool down, 10′ resistance exercise, 5′ relaxation	4 months	SDS ventriculography: rest LVEF peak LVEF peak emptying rate (PER) peak filling rate (PFR)	↑ [16] Patients: abnormal LVEF/PER response. Post-training LVEF unchanged; no correlation between LVEF in SDS

*angio* coronary angiogram, *BMI* body mass index, *CAD* coronary artery disease, *CP* chest pain, *EST* exercise stress test, *ET* exercise training, *LVEF* left ventricular ejection fraction, *nl* normal, *ns* non-significant, *NTG* nitroglycerine, *QoL* quality of life, *SDS* summed difference score, *SSS* summed stress score, *SRS* summed rest score, *SPECT* single photon emission computed tomography, *SBP/DBP* systolic/diastolic blood pressure, *sx* symptoms, *wk* week, *WR* work rate

## Discussion

Review of the current literature on effects of physical exercise in NOCAD patients with angina yielded a limited number of studies. ET appears to be beneficial in patients with CSX/microvascular angina who are notoriously difficult to treat with one third remaining symptomatic in spite of treatment [5]. Albeit training protocols differed between the study populations, moderate activity 2–3 times per week (minimum of 90 min/week) appears to improve physical functioning over 8 weeks. This is of importance since impaired physical functioning is a feature of symptomatic patients with NOCAD [7]. Furthermore, Jespersen et al. showed that severity of persistent angina is associated with impaired physical functioning and QoL in NOCAD patients [7]. Asbury et al. demonstrated that ET reduced the severity of pain episodes, which may partially explain the improved QoL [15]. It remains uncertain whether this is mediated by an increased myocardial perfusion through improved coronary endothelial function. None of the studies looked at coronary endothelial function directly. One study showed a trend towards improvement of peripheral endothelial function using FMD, despite the fact that the study was underpowered [13]. There is also one case report which supports this hypothesis by showing a reversal of RPDs together with an improved peripheral endothelial function [20]. Furthermore, studies in diabetic patients with CAD showed that coronary endothelial function is improved by ET, which might explain the positive effects observed in the NOCAD population [21].

Although a variety of exercise protocols seem to have a beneficial effect on symptomatic patients with NOCAD, the optimal training strategy and guidance remain uncertain. Carvalho et al. used a longer duration of the CR program (4 months) but did not adapt the training intensity over the course of the program and patients had only a mild increase in VO_2_ of 13% [16]. The authors were not able to show an improvement in general health status as assessed by SF-36. This may be related to the limited training effect. Another possible explanation is that the SF-36 questionnaire is a well-established generic questionnaire, which evaluates parameters like general health perception, social functioning, and mental health [22]. Application of a more disease-specific questionnaire such as the Seattle Angina questionnaire might have yielded different results [23].

As recommended by guidelines, authors chose mostly a moderate training intensity, although guidance parameters varied between the studies. A meta-analysis recently showed that high-intensity training improves endothelial function in patients with cardiovascular disease [24], hereby raising the question whether it might be a time-efficient alternative or addition in NOCAD patients. Furthermore, training strategies like skiing appear to have a beneficial effect on endothelial health, which might broaden the recommended exercise spectrum, at least in alpine regions [25].

The reported trials have several limitations. Firstly, they were all small with low patient numbers in each treatment group, hereby limiting statistical power. In addition, not all of the studies were randomized. Secondly, the majority of studies included only women (97.7%). Although cardiac syndrome X is more common in women, it is well established that it also occurs in men, with up to 30% of men with SA presenting for coronary angiogram, have NOCAD [1••]. Given that the studies were limited to women, we can only speculate whether ET has the same positive effect in men.

Outcome measures in the reported trials consisted mostly of parameters for exercise capacity, easily measurable physical values, and QoL assessed by questionnaires. All outcomes were evaluated in the short term, directly after completion of the CR program. No data is available on the long-term effects of CR programs in NOCAD, and whether the beneficial effect is sustained over time. Furthermore, it would be interesting whether this transfers into hard endpoints like less frequent hospitalization, lower treatment costs, and possibly an improved outcome. For a long time, symptomatic patients with NOCAD were assured of the benign nature of their condition. However, recent data points towards an adverse outcome of these patients in regard to myocardial infarction, cardiovascular, and all-cause mortality [1••, 26]. Therefore, it would be intriguing to assess whether CR also leads to an improved cardiovascular outcome in this patient population.

### Future research

Current studies on the effect of ET in symptomatic patients with NOCAD are promising but larger, randomized studies with inclusion of male patients are needed to evaluate the benefit of ET on hard endpoints and the long-term effect of ET. Furthermore, a study protocol should include randomized groups to determine the optimal training protocol in regard to training intensity, duration, and inclusion of relaxation techniques. Furthermore, it would be of interest to include vascular function studies to gain further insight into the pathophysiological mechanisms.

## Conclusion

Available evidence suggest that exercise training in women with angina and NOCAD has a beneficial effect on exercise capacity, severity of symptoms, and quality of life and appears to improve myocardial perfusion defects. Further, larger trials with inclusion of men are warranted in this challenging population, which is commonly subjected to numerous medical and invasive therapies of uncertain efficacy.
